# Slp1 and Slp2-a Localize to the Plasma Membrane of CTL and Contribute to Secretion from the Immunological Synapse

**DOI:** 10.1111/j.1600-0854.2008.00714.x

**Published:** 2008-02-11

**Authors:** Oliver Holt, Eiko Kanno, Giovanna Bossi, Sarah Booth, Tiziana Daniele, Alessandra Santoro, Maurizio Arico, Chika Saegusa, Mitsunori Fukuda, Gillian M Griffiths

**Affiliations:** 1 Sir William Dunn School of Pathology, University of Oxford South Parks Rd, Oxford, OX1 3RE, UK; 2 Laboratory of Membrane Trafficking Mechanisms, Department of Developmental Biology and Neurosciences, Graduate School of Life Sciences, Tohoku University Aobayama, Aoba-ku, Sendai, Miyagi 980-8578, Japan; 3 Fukuda Initiative Research Unit, RIKEN (The Institute of Physical and Chemical Research) 2-1 Hirosawa, Wako, Saitama 351-0198, Japan; 4 Current address: Cambridge Institute for Medical Research Wellcome/MRC Building, PO Box 139, Addenbrooke's Hospital, Hills Road, Cambridge CB2 0XY, UK; 5 Azienda Ospedaliero-Universitaria Meyer Via Luca Giordano, 13, Viale Pieraccini, 24 50100 Firenze, Italy

**Keywords:** CTL, immunological synapse, Rab27a, secretory lysosome, Slp2-a

## Abstract

Rab27a is required for polarized secretion of lysosomes from cytotoxic T lymphocytes (CTLs) at the immunological synapse. A series of Rab27a-interacting proteins have been identified; however, only Munc13-4 has been found to be expressed in CTL. In this study, we screened for expression of the synaptotagmin-like proteins (Slps): Slp1/JFC1, Slp2-a/exophilin4, Slp3-a, Slp4/granuphilin, Slp5 and rabphilin in CTL. We found that both Slp1 and Slp2-a are expressed in CTL. Isoforms of Slp2-a in CTL showed variation of the linker region but conserved the C2A and C2B and Slp homology (SHD) domains. Both Slp1 and Slp2-a interact with Rab27a in CTL, and Slp2-a, but not Slp1, is rapidly degraded when Rab27a is absent. Slp2-a contains PEST-like sequences within its linker region, which render it susceptible to degradation. Both Slp1 and Slp2-a localize predominantly to the plasma membrane of both human and mouse CTLs, and we show that Slp2-a can focus tightly at the immunological synapse formed with a target cell. Individual knockouts of either Slp2-a or Slp1 fail to impair CTL-mediated killing of targets; however, overexpression of a dominant-negative construct consisting of the SHD of Slp2-a, which is 56% identical to that of Slp1, reduces target cell death, suggesting that both Slp1 and Slp2-a contribute to secretory lysosome exocytosis from CTL. These results suggest that both Slp1 and Slp2-a may form part of a docking complex, capturing secretory lysosomes at the immunological synapse.

Cytotoxic T lymphocyte (CTL) cells use polarized secretion of lysosomal organelles to destroy virally infected and tumorigenic cells. Secretion occurs at a precise point within the immunological synapse formed between CTL and target cell (1). The mechanism of secretion is unusual: recognition of the target by the T-cell receptor triggers centrosomal polarization to a precise location within the immunological synapse, next to the central supramolecular activation complex (2,3). Secretory lysosomes move along the microtubules in the minus direction towards the microtubule-organizing centre (MTOC) and are delivered as the centrosome ‘docks’ at the membrane.

Both Rab27a and its binding partner Munc13-4 are essential for lysosomal secretion from CTL, and loss of either Rab27a (4,5) or Munc13-4 (6) leads to loss of secretion, with the granules polarized towards the immunological synapse but unable to undergo secretion. Rab27a also plays a key role in secretory lysosome secretion in a number of cell types with secretory lysosomes or related organelles including melanocytes (7,8), neutrophils (9) and pancreatic beta cells (10). In each of these cell types, Rab27a mediates secretion by interacting with different effector proteins: in melanocytes, Rab27a mediates transfer of lysosome-related melanosomes from the plus ends of microtubules to the actin cytoskeleton by interactions with Slac2-a/melanophilin and myosin Va (reviewed in 11). Rab27a also mediates anchoring of melanosomes to the plasma membrane through interaction with synaptotagmin-like protein (Slp)2-a (12). In neutrophils, Rab27a interacts with Slp1/JFC1 (9), and in pancreatic alpha and beta cells, Rab27a interacts with Slp2-a/exophilin4 (13) and Slp4/granuphilin (14), respectively. These interactions are thought to promote the targeting of the granules towards the plasma membrane, possibly by mediating docking.

A family of six different Rab27a-binding Slps have been identified, all of which contain an N-terminal Slp homology domain (SHD), which binds Rab27a in a GTP-dependent manner, a central linker region and two C2 domains capable of binding to phospholipid membranes (15). In this paper, we ask whether Slp proteins are expressed in CTL. We find expression of Slp1 and Slp2-a, both of which bind to Rab27a in CTL. Slp2-a is readily degraded because of the presence of PEST-like sequences, and in the absence of Rab27a, Slp2-a protein levels are also reduced. Slp2-a associates with the plasma membrane and focuses at the immunological synapse formed between CTL and target. Expression of dominant-negative Slp2-a, lacking the C2 domains, reduces CTL-mediated killing. These results suggest that Slp2-a may form part of the docking complex, which tethers secretory lysosomes to the plasma membrane as they reach the immunological synapse.

## Results

### Slp1 and novel isoforms of Slp2-a are expressed in CTL

**Figure 1:**
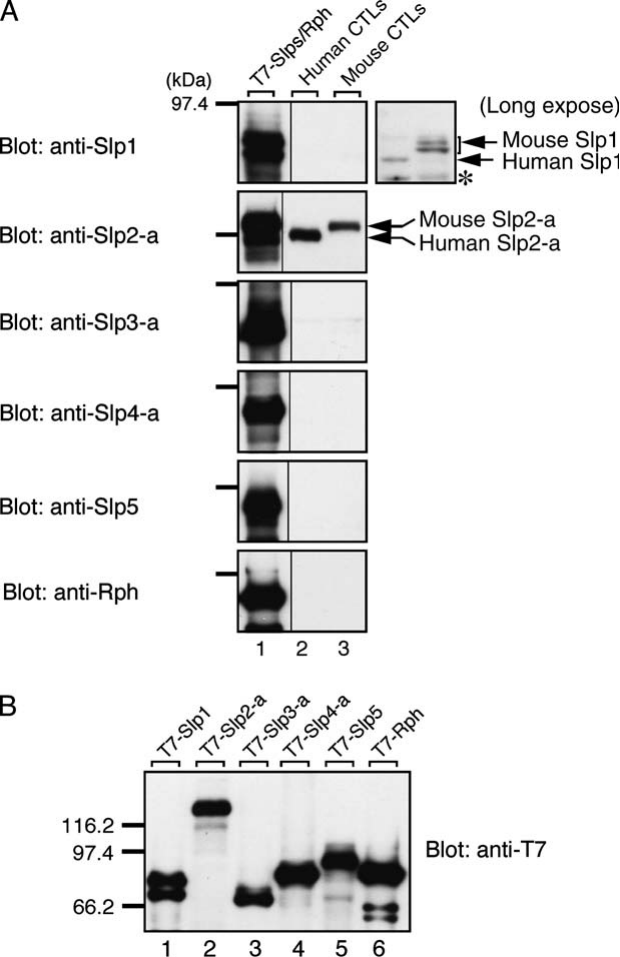
Expression of Slp1 and Slp2-a in CTL A) Western blots showing mouse and human CTL lysates probed for expression of Slp1-5 and rabphilin (Rph). Lanes show control lysates of T7-tagged Slp1-5 or Rph (lane 1) and lysates from human (lane 2) and mouse CTLs (lane 3). Blots are probed with antibodies against Slp1-5 and Rph as described in Imai et al. (37). The marker on the left shows the relative migration of the 97.4 kDa molecular weight marker. A longer exposure of Slp1 is shown on the right. Note that the Slp1 and Slp2-a proteins, but not the other Slp proteins, were detected in human and mouse CTLs (indicated by arrows). Asterisk corresponds to non-specific bands. B) Recombinant T7-tagged Slp1-5 and Rph were used as positive controls in A. Similar amounts of the T7-tagged proteins from COS-7 cell lysates were loaded into each lane and probed with anti-T7 tag antibody as described previously (37). Relative migration of molecular weight markers (in kiloDalton) is shown on the left.

Cell lysates from human and mouse CTLs were screened for expression of Slps by Western blotting (Figure 1). Antibodies against Slp1 SHD revealed bands in both human and mouse CTLs lysates. In human CTL lysates, a dominant band with an apparent molecular weight of 58 kDa was present, and in mouse, two bands of apparent molecular weights of 60 and 62 kDa were seen (Figure 1A). Because no alternative splice form of Slp1 has been reported and complementary DNA (cDNA) cloning from CTL produced only the reported isoform, it is possible that the upper bands may correspond to either a post-translationally modified form of Slp1 or a cross-reactive protein. A faint upper band and the lower band marked with an asterisk are non-specific. Antibodies against either Slp2-a SHD (Figure 1A) or linker region (not shown) revealed single strong bands of 97 kDa in human and 100 kDa in mouse CTL lysates.

**Figure 4:**
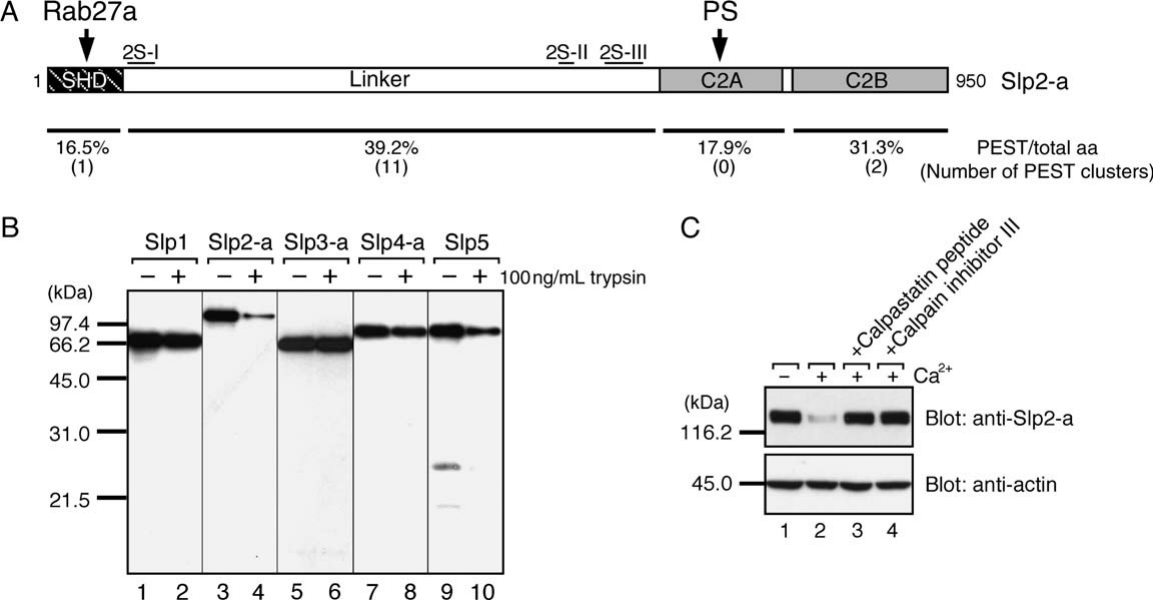
Slp2-a is readily degraded by proteases and endogenous calpains A) Scheme to show the PEST sequence distribution in the 950 amino acid Slp2-a sequence encoded by full-length mouse Slp2-a (accession number: AB057754), indicating the SHD that binds Rab27a (15), C2A that binds phosphatidylserine (PS) (12) and C2B domains. The sites where splicing variation within the linker have been reported are shown, 2S-I, 2S-II and 2S-III, as well as the content of four amino acids (Pro, Glu, Ser and Thr) in each domain and the number of clusters of PEST-like sequences identified by eye. B) COS-7 cell lysates expressing T7-tagged Slp1-5 treated with (+) or without (−) 100 ng/mL trypsin and probed with anti-T7 antibody. C) Cell lysates from melanocytes expressing Slp2-a treated with calpastatin peptide or calpain inhibitor in the presence of calcium (+) and probed with anti-Slp2-a or anti-actin antibody. Relative migration of molecular weight markers (in kiloDalton) is shown on the left.

We have previously reported three different isoforms of Slp2-a generated from differential splicing, which have different expression patterns in different mouse tissues (16). In order to determine which splice forms of Slp2-a are expressed in mouse CTL, we cloned and sequenced cDNAs corresponding to Slp2-a from mouse CTL. We found two splice forms of Slp2-a in CTL derived from C57BL/6 mice. The first form is the previously described 2S-II isoform (accession number: AB057761), which lacks 16 amino acids (539–554) from the linker region of Slp2-a. The second form, which combines the deletions seen in the 2S-II and 2S-III isoforms previously described (accession numbers: AB057761 and AB057762), lacks both the 2S-II site (amino acids 539–554) and the 2S-III site (amino acids 587–626) from the linker region of Slp2-a. Except for Figure 4, all subsequent experiments in this study were carried out with the latter splice variant, the sequence of which is given in Figure S1. The SHD and C2 domains are conserved in both variants.

### Slp1 and Slp2-a bind to Rab27a in CTL lysates

**Figure 2:**
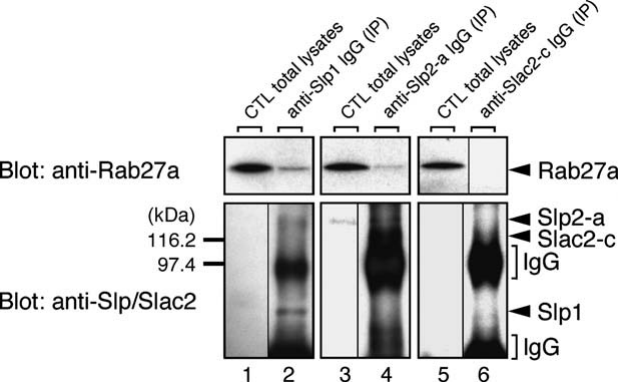
Slp1 and Slp2-a interact with Rab27a from CTL lysates Western blots of mouse CTL total lysates (lanes 1, 3 and 5) or immunoprecipitated with antibodies against Slp1 (lane 2), Slp2-a (lane 4) or Slac2-c (lane 6), probed with anti-Rab27a or anti-Slp/Slac2 antibody. Immunoprecipitation with anti-Slp1, anti-Slp2-a or control anti-Slac2-c IgG was performed as described in the *Materials and Methods*. Coimmunoprecipitated Rab27a was detected by immunoblotting with anti-Rab27a antibody (indicated by arrowheads; lanes 2, 4 and 6 in the top panel). Immunoprecipitated Slp1, Slp2-a and Slac2-c were then visualized by immunoblotting with specific antibodies (indicated by arrowheads; lanes 2, 4 and 6 in the bottom panel). Non-specific bands of IgG used for immunoprecipitation are indicated by IgG. Note that both anti-Slp1 and anti-Slp2-a IgGs, but not Slac2-c IgG, immunoprecipitated Rab27a (lanes 2 and 4 in the top panel). Under our experimental conditions, anti-Slp1 IgG immunoprecipitated Slp1 protein (and also Rab27a protein) more efficiently than anti-Slp2-a IgG did Slp2-a protein. Relative migration of molecular weight markers (in kiloDalton) is shown on the left. IP, immunoprecipitate.

Both Slp1 and Slp2-a have been shown to bind to Rab27a, a protein that is required for CTL secretion. In order to demonstrate that Slp1 and Slp2-a were able to interact with Rab27a, mouse CTL lysates were precipitated with antibodies against Slp1, Slp2-a and the related protein, Slac2-c, which is not expressed in CTL. Western blots were then probed with antibodies against Rab27a and the Slp1, Slp2-a or Slac2-c (Figure 2). Immunoprecipitation with Slp1 and Slp2-a both co-precipitated Rab27a, demonstrating that Rab27a is able to interact with Slp1 and Slp2-a in CTL (lanes 2 and 4 in the top panel). By contrast, Slac2-c could not be precipitated from CTL lysates (lane 6 in the top and bottom panels).

**Figure 3:**
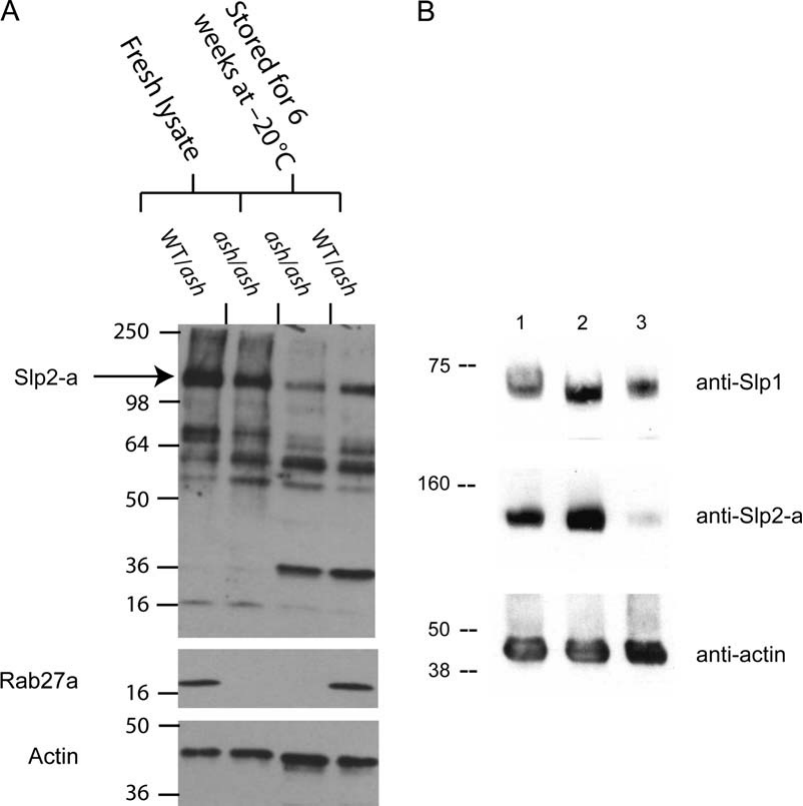
Slp2-a is readily degraded and stabilized by association with Rab27a A) Western blots of mouse CTL lysates from heterozygous or homozygous *ashen* mice prepared and run on SDS–PAGE immediately (fresh lysate) or stored for 6 weeks at −20°C before analysis. The nitrocellulose filter was probed with anti-Slp2-a antibody, washed and reprobed with anti-Rab27a and anti-actin antibodies. WT, wild type. B) Western blots of human CTL lysates derived from healthy donors (lane 1) and patients lacking Munc13-4 (lane 2) or Rab27a (lane 3) probed with antibodies to Slp1 SHD, Slp2-a SHD or actin. Relative migration of molecular weight markers (in kiloDalton) is shown on the left.

Cell lysates from *ashen* mice that lack Rab27a show decreased levels of Slp2-a, which can be observed both in fresh lysates and more noticeably in lysates that have been frozen and thawed (Figure 3A). Actin controls show equal levels for CTL lysates derived from *ash/ash* or *ash*/+ mice regardless of whether lysates were prepared freshly or whether they had been frozen and thawed. Rab27a is also present at equivalent levels in both fresh and freeze-thawed lysates from *ash*/+ CTL. Slp2-a appears to be highly susceptible to degradation, with the band of approximately 100 kDa molecular weight strongest in the *ash*/+ fresh (lane 1) compared with freeze-thawed lysate (lane 4) in which a degradation product of 36 kDa is seen. Slp2-a levels are also affected by the presence of Rab27a as both fresh and frozen lysates show increased levels of Slp2-a in *ash*/+ lysates compared with *ash/ash*lysates. This is particularly noticeable once samples have been frozen (lanes 3 and 4).

Similar results are seen in cell lysates from human CTL lacking Rab27a (Figure 3B). CTL lysates derived from healthy (lane 1), Munc13-4-deficient (lane 2) and Rab27a-deficient (lane 3) cells were probed with antibodies to Slp1, Slp2-a and actin. Munc13-4 and Rab27a mutations were confirmed by genomic sequencing, and both cDNA and genomic sequencing were used to confirm that there were no mutations present in Slp2-a in the Rab27a-deficient patient. While actin levels are comparable between lanes 1–3, the signal for Slp2-a is markedly decreased in the cell lysate lacking Rab27a. Protein levels for Slp1 do not decrease in the lysate from cells lacking Rab27a.

These results suggest that Slp2-a is highly susceptible to degradation and that it is stabilized by interaction with Rab27a. Similar stabilization of Slac2-a/melanophilin, another Rab27a effector in melanocytes, by interaction with Rab27a has been reported in *ashen* melanocytes (17). Interestingly, Slac2-a/melanophilin has recently been shown to be readily degraded by several proteases, including endogenous calpains in melanocytes (18), because it contains potential PEST sequences, which were originally identified (19,20) as motifs that target proteins for increased susceptibility for degradation (21). We therefore attempted to investigate whether Slp2-a also contains PEST-like sequences and inspected its primary sequences.

### Slp2-a contains PEST-like sequences and is readily degraded

Four separate domains have been identified in Slp2-a (Figure 4A): the SHD, which binds Rab27a, the linker region and the phospholipid-binding C2A and C2B domains. Each of these domains was examined for the presence of PEST-like sequences [containing the amino acids proline (P), glutamic acid (E), serine (S) and threonine (T)]. A bioinformatic search of the SHD, linker and C2 domains of murine Slp2-a identified 14 different PEST-like clusters, with 1 in the SHD, 11 in the linker and 2 in the C2B domain. In order to test whether Slp2-a showed increased susceptibility to degradation, T7-tagged Slp1-5 proteins were expressed in COS-7 cells and cell lysates were prepared in the absence of protease inhibitors and incubated at 25°C for 30 min with or without 100 ng/mL of trypsin before immunoblotting and probing with antibodies against T7 tag (Figure 4B). Slp1 and Slp3-a showed no decrease in the intensity of the protein band in the presence of trypsin, while Slp4-a and Slp5 showed a modest reduction. By comparison, Slp2-a showed a dramatic reduction in the amount of protein detected after trypsin treatment *in vitro*.

In order to assess whether Slp2-a might be degraded by endogenous calpains in a calcium-dependent manner, total cell lysates of melanocytes were prepared in the absence of protease inhibitors. Cell lysates were incubated at 30°C for 1 h with either 2 mm EGTA or 750 μm Ca^2+^ in the presence or absence of calpain inhibitors and probed with anti-Slp2-a antibody. Slp2-a is readily degraded compared with the actin controls only in the presence of calcium. Both calpastatin peptide and calpain inhibitor III are able to prevent the calcium-mediated degradation (Figure 4C). These results demonstrate that Slp2-a might be degraded endogenously in response to calcium-dependent calpains, suggesting that rapid degradation may provide a way for regulating Slp2-a activity.

### Slp1 and Slp2-a are localized on the plasma membrane and can focus at the immunological synapse

**Figure 5:**
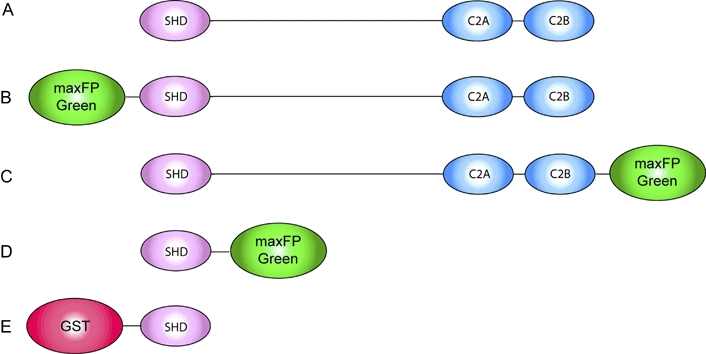
Scheme of constructs used in this study Schematic diagram showing the domains of Slp1 or Slp2-a with the SHD at the amino terminus and two C2 domains at the carboxy terminus (A). Constructs tagging either N-terminus (B) or C-terminus (C) were used in this study as was a dominant-negative construct using just the SHD fused to maxFP Green (pmaxGFP) (D). A glutathione S-transferase fusion protein of the SHD (E) was used to produce antibodies.

**Figure 6:**
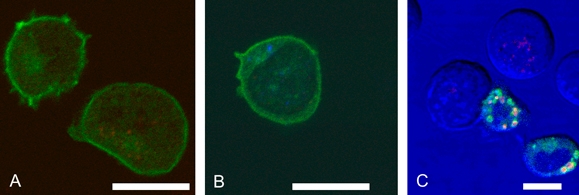
Slp1 and Slp2-a localize to the plasma membrane in CTL Activated human CD3 cells were transfected with A) human Slp1–EGFP and co-stained with granzyme A (red) and B) human Slp2-a–EGFP and co-stained with cathepsin D (blue). C) CTL derived from transgenic mice expressing Rab27a–EGFP and co-labelled with Lysotracker Red to show lysosomes recognize P815 targets (unlabelled). Scale bars: 10 μm.

Antibodies raised against Slp1 and Slp2-a are unable to detect endogenous protein in CTL by immunofluorescence. We therefore tagged Slp1 and Slp2-a at N- or C-termini with either enhanced green fluorescent protein (EGFP) or maxFP Green (pmaxGFP) and expressed these constructs in human and mouse CTLs (Figure 5). Slp1 and Slp2-a tagged with EGFP at the amino terminal end of the protein (Figure 5A) were expressed in activated human T cells (Figure 6), and both Slp1 (Figure 6A) and Slp2-a (Figure 6B) showed strong association with the plasma membrane. There was no obvious overlap with the granule stainings shown by granzyme A (red, A) or cathepsin D (blue, B). This suggests that the dominant localization of both Slp1 and Slp2-a is at the plasma membrane. This differs markedly from the localization of Rab27a in CTL, which localizes to the lytic granules (22). Figure 6C and Movie S1 show two CTLs derived from a transgenic mouse expressing Rab27a–green fluorescent protein (GFP) under the control of the Rab27a promoter (22). One CTL is conjugated to a P815 target cell, and both are loaded with Lysotracker Red to mark the lysosomes. Some cytosolic Rab27a is visible, but the majority is closely associated with the lytic granules, both near the immunological synapse and at the rear of the CTL as well as in the unconjugated CTL in the bottom right of the Movie S1.

**Figure 7:**
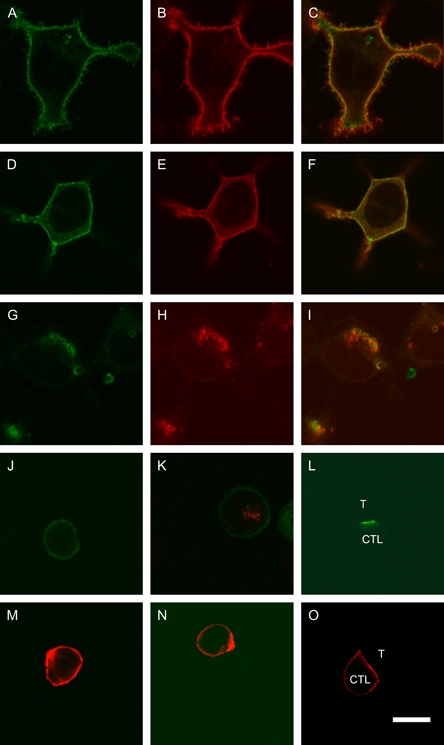
Slp2-a localizes predominantly to the plasma membrane and focuses at the immunological synapse Mouse Slp2-a tagged with pmaxGFP at either the amino terminus (panels A–C) or the carboxy terminus (panels D–I) expressed in RBL cells (panels A–I). Panels A, D and G show GFP fluorescence, and B and E show staining with anti-Slp2-a SHD antibody. Panel H shows Lgp100 staining. Panels C, F and I are merged images of A and B, D and E, and G and H, respectively. Panels J–O show N-terminally pmaxGFP-tagged mouse Slp2-a expressed in mouse CTL either unstained (panels J and L) or counterstained with anti-Lamp2 antibody ABL-93 (panel K) or anti-Slp2-a SHD antibody (panels M–O). Letter T indicates P815 target cell. All panels to the same scale. Scale bar: 10 μm.

Because the initial constructs were both tagged at the amino terminus, next to the SHD where Rab27a binds, we asked whether the localization might differ if tagged at the C-terminus (Figure 5C). We therefore expressed Slp2-a tagged at the N- and C-termini in the rat basophilic cell line, RBL, as this cell line is easily transfectable and possesses secretory lysosomes to which both Rab27a and Munc13-4 will localize correctly (23). Both Slp2-a tagged at the N- (Figure 7, panels A–C) and C-termini (Figure 7, panels D–F) gave a predominant localization to the plasma membrane; however, in some cells, granule localization could be seen when Slp2-a was tagged at the C-terminus (Figure 7, panels G–I), as Slp2–a GFP colocalized with anti-Lgp100 staining (I). We confirmed the specificity of our anti-Slp2-a SHD-purified rabbit antibody in RBL cells transfected with Slp2-a tagged at both ends (B, E and H).

We then asked whether Slp localization changed upon formation of the immunological synapse by expressing Slp1 and Slp2-a in mouse CTL and examining localization in single and conjugated cells. Expression of Slp1 was not detectable, but Slp2-a tagged at the N-terminus could be detected by pmaxGFP expression (J–L) and enhanced by staining with the anti-Slp2-a SHD antibody (M–O). In isolated CTL (J, K, M and N), Slp2-a is localized around the plasma membrane and did not colocalize with lysosomes (Figure 7, panel K). In conjugated CTL, Slp2-a focuses at the immunological synapse (L and O).

### Single knockouts of Slp1 and Slp2-a do not interfere with killing, but a dominant-negative Slp2-a SHD reduces CTL-mediated killing of target cells

**Figure 8:**
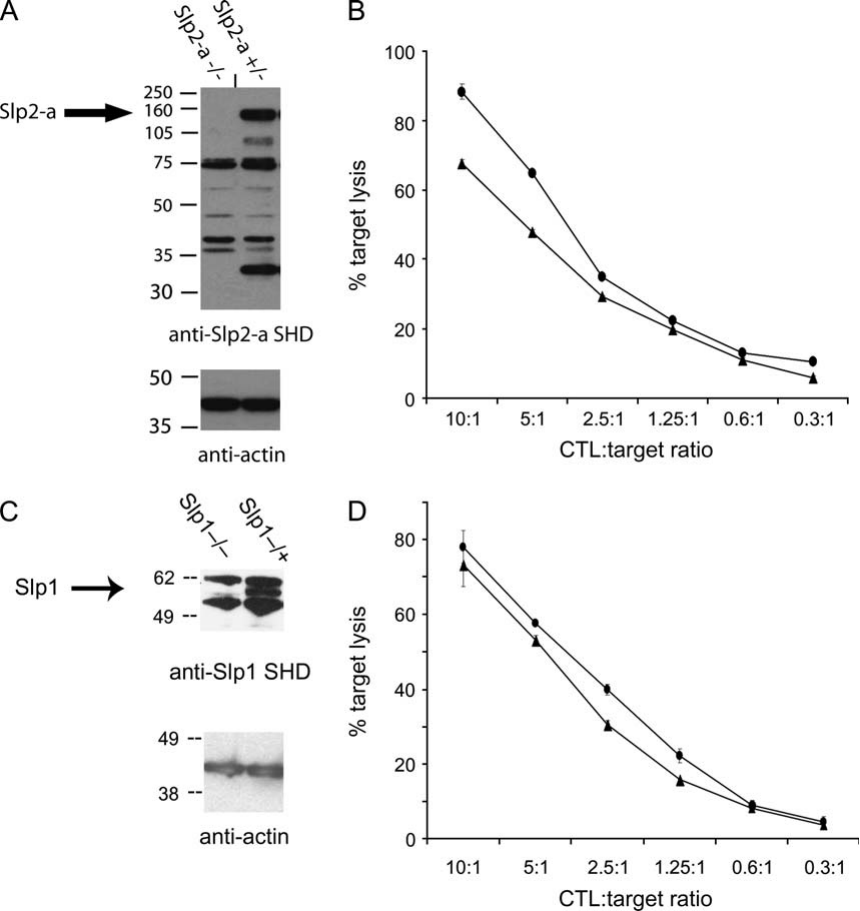
Slp2-a and Slp1-deficient CTL show normal levels of killing A) Western blots of CTL lysates probed with anti-Slp2-a SHD or actin antibodies and B) killing assays from Slp2-a −/− (triangles) and +/− (circles) mice. C) Western blots probed with anti-Slp1 SHD or actin antibodies and D) killing assays showing percentage target cell lysis from Slp1 +/− (circles) and Slp1 −/− (triangles). Error bars represent standard deviation from triplicates for each CTL:target ratio. Molecular weight markers shown on the left of Western blots (in kiloDalton) with arrows pointing to the bands corresponding to Slp2-a and Slp1.

In order to ask whether Slp1 and Slp2-a might play a role in killing, we examined the ability of CTL generated from Slp1 −/− and Slp2-a −/− mice (24) to kill targets. The absence of protein from CTL generated from Slp2-a −/− mice was confirmed by Western blotting using an antibody against Slp2-a SHD while actin levels were equivalent (Figure 8A). CTL generated from both Slp2-a +/− and −/− mice killed targets well with no significant reduction of killing from that of the Slp2a-deficient CTL (Figure 8B), suggesting that if Slp2-a is involved in killing, there may be functional redundancy with other protein(s) such as Slp1. We therefore examined the ability of CTL from recently generated Slp1-deficient mice (C. Saegusa, S. Itohara and M. Fukuda, manuscript in preparation) in which insertion of neomycin was used to replace exon 1 of Slp1. Western blotting of CTL lysates with anti-Slp1 SHD antibody produced three bands of 58, 60 and 62 kDa of which the 60 kDa was specifically missing in CTL from Slp1 −/− mice (Figure 8C), suggesting that this band corresponds to the single isoform of Slp1 detected by cDNA cloning. Actin levels were equivalent in Slp1 −/− and +/− lysates. CTL from Slp1 −/− and Slp1 +/− mice showed similar levels of killing (Figure 8D), suggesting that loss of Slp1 alone was insufficient to disrupt killing.

**Figure 9:**
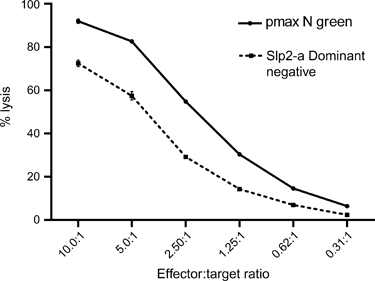
Expression of a dominant-negative SHD from Slp2-a reduces CTL-mediated killing Killing assay showing percentage lysis of targets at differing ratios of effector CTL to target in CTL expressing pmaxGFP (bold line) or the dominant-negative construct (broken line) expressing the SHD of Slp2-a tagged with pmaxGFP. Error bars show standard deviation from triplicates.

We therefore generated a dominant-negative construct consisting of the SHD of Slp2-a fused to GFP. Because the SHDs of Slp1 and Slp2-a are 56% identical and 73% similar in amino acid sequence, we reasoned that overexpression of the dominant-negative construct might interfere with Rab27a binding of both endogenous Slp1 and Slp2-a. CTLs were transfected with dominant-negative SHD–GFP, and transfected cells were sorted for GFP expression before assaying killing of P815 target cells. Target cell killing was reduced, although not ablated (Figure 9), suggesting that Slp1 and Slp2-a together play a positive regulatory role in CTL killing.

## Discussion

Rab27a has been shown to play a key role in secretion from both CTL and melanocytes. Loss of Rab27a prevents secretion from both melanocytes and CTL leading to Griscelli syndrome with albinism and immunodeficiency (4). In melanocytes, the series of protein interactions leading to melanosome secretion is well understood, with Rab27a on the melanosomes interacting with Slac2-a/melanophilin that in turn enables a tripartite complex to form with myosin Va, which can transfer the melanosomes from the microtubule to the actin cytoskeleton (reviewed in 11). Rab27a, Slac2-a/melanophilin and myosin Va are all required for melanosome secretion, but neither Slac2-a/melanophilin nor myosin Va is required for secretion from CTL.

Not only do the proteins involved in mediating secretion differ between CTL and melanocytes but also the mechanics of secretion differ. Melanosomes move from a pericentriolar localization towards the periphery in a plus-end direction on microtubules, where they are captured onto the cortical actin cytoskeleton by myosin Va (11). Secretion from CTL is almost the reverse of this, with the centrosome of the MTOC moving to the point of secretion at the immunological synapse and the lytic granules moving in a minus-end direction towards the MTOC and the site of secretion (2). The actin cytoskeleton is cleared away from the point of secretion, and the granules appear to be delivered to the plasma membrane directly from microtubules. Although Rab27a is required for both melanosome and lytic granule secretion, it has become clear that Rab27a is interacting with different effector proteins in order to accomplish this.

Eleven Rab27a-interacting proteins have been identified in mouse and man (25,26). Ten of these are Slps (Slp1/JFC1, Slp2-a/exophilin4, Slp3-a, Slp4/granuphilin, Slp5, rabphilin, Slac2-a/melanophilin, Slac2-b, Slac2-c/MyRIP and Noc2) containing SHD-Rab27a-binding domains. The eleventh is Munc13-4, which contains a novel Rab27a-binding domain distinct from the SHD (23,27). Munc13-4 is required for granule exocytosis from CTL, and granules in CTL deficient in Munc13-4 are found docked, but unable to fuse, at the plasma membrane in the immunological synapse (6,28). Munc13-4 is thought to act as a priming protein for SNAREs by analogy to the neuronal protein Munc13-1, which is thought to hold t-SNAREs in an open conformation and act as a priming factor for membrane fusion (29,30), although the details of its role are yet to be fully defined (reviewed in 31).

Both Munc13-4 and Rab27a have been localized to the lytic granules, and it is not clear how the granules are passed to the plasma membrane prior to docking and secretion. Slp4/granuphilin has been shown to be involved in docking insulin granules to the fusion machinery at the plasma membrane, and mice deficient in Slp4/granuphilin show fewer granules docked at the plasma membrane even though exocytosis is enhanced (14), suggesting that Slp4/granuphilin plays a negative regulatory role in exocytosis (14,32).

In the present study, we have examined the expression of Slp proteins in CTL and find expression of Slp1 and Slp2-a in both mouse and human CTLs. The same expression pattern is found in human natural killer cells (data not shown). Cloning cDNAs from CTL, we found that the different splice forms generated from the 19 exons conserved the SHD and C2 domains but showed a novel pattern of deletion within the linker region, the function of which remains unknown. We found a similar picture in cDNA cloned from human CTL where we identified multiple splice variants within the region encoding the linker region, but in each case, the SHD and C2 domains were present. Only a single isoform of Slp1 has been identified (33), and cDNA cloning from both mouse and human CTLs identified only this isoform. Our Western blots using the anti-Slp1 SHD antibody showed only a single clear band reproducibly detected from human CTL lysates (Figures 1 and 3B) but three bands from mouse CTL lysates (Figures 1 and 8C). Because there is only one isoform detected at the cDNA level and only the 60-kDa band disappears in CTL derived from an Slp1-null mouse, it seems most likely that only the 60-kDa band represents murine Slp1. We do not know what the 58 and 62 kDa cross-reactive bands represent.

Both Slp1 and Slp2-a bind Rab27a in CTL. However, in CTL derived from mice lacking Rab27a, we noted that Slp2-a, but not Slp1, was less stable (Figure 3). This loss of stability of Slp2-a, but not Slp1, in the absence of Rab27a most likely arises from the presence of multiple PEST-like sequences, which render it readily degraded (Figure 4). Similar PEST-like sequences have been identified in Slac2-a/melanophilin (18). Studies in melanocytes suggested that this overexpression might have a functional role as overexpression of a mutant form of Slac2-a/melanophilin lacking the PEST-like sequences resulted in the increased rate of perinuclear aggregation of melanosomes. This suggested that endogenous degradation of Slac2-a/melanophilin may be important for releasing the melanosomes from the microtubule cytoskeleton to the actin cytoskeleton. These results suggest that Slp2-a is stabilized by its interaction with Rab27a and that this may have functional consequences *in vivo*.

Expression of Slp1 and Slp2-a in CTL showed that both localized predominantly to the plasma membrane (Figure 7). Because these experiments were carried out with Slp2-a tagged at the N-terminus that might interfere with membrane association by the Rab27a-binding SHD, we tagged Slp2-a at both N- and C-termini to investigate membrane localization in RBL cells, which also express Rab27a (34). Using a C-terminally tagged Slp2-a, the predominant membrane association remained at the plasma membrane, although a rare cell could be found with lysosomal-associated Slp2-a. In CTL, endogenous Slp2-a could not be detected by immunofluorescent labelling. This is consistent with previous studies that have shown that neither endogenous Rab27a nor Munc13-4 can be detected in CTL (22,35) and presumably reflects a relatively low level of expression of these proteins in CTL. We therefore expressed both Slp1 and Slp2-a fused to pmaxGFP in CTL and found that both localized to the plasma membrane. Slp2-a–GFP localized to the plasma membrane of CTL whether tagged at the N- or C-terminus, although the N-terminally tagged construct was expressed more efficiently in all cell lines tested. This might reflect membrane localization by the C2A and C2B domains, which may be hindered when pmaxGFP is fused to the carboxy terminus of the protein close to the C2 domains. Strikingly, Slp2-a focussed at the point of contact between CTL and target, within the immunological synapse, suggesting that this protein might play an important role in granule exocytosis from CTL. It is possible that Slp1 also focuses at the immune synapse, but expression of this construct in mouse CTL was inefficient, and we were unable to see transfected conjugates.

How might Slp2-a function? The membrane localization we observed in CTL for Slp1 and Slp2-a and the clustering of Slp2-a in the immunological synapse suggest that Slp1 and Slp2-a may act as part of a docking complex which, by interaction with Rab27a on the lytic granules, might serve to capture the granules as they reach the plasma membrane and dock them prior to secretion. Slp2-a is involved in mucus secretion by gastric surface mucous cells because a deficiency of granule docking and reduction of mucus secretion were observed in Slp2-a −/− mice (24). In addition, Slp2-a is thought to target glucagon granules to the plasma membrane in pancreatic alpha cells because overexpression of Slp2-a containing mutations that prevent C2A binding to phospholipid membranes inhibits evoked glucagon secretion from αTC1.6 cells (13). More recently, the C2 domains of synaptotagmins have been found to be important for producing membrane curvature and have been proposed to provide a general mechanism for promoting SNARE fusion (36). Virtually, all C2 domains from synaptotagmins tested induced membrane curvature in a calcium-dependent manner, with the exception of Slp2-a that was unaffected by calcium.

Our functional studies using knockout mice for Slp1 and Slp2-a revealed normal levels of CTL killing of target cells, suggesting that loss of either Slp1 or Slp2-a alone is insufficient to impair granule secretion from CTL. We therefore used a dominant-negative construct consisting of the SHD from Slp2-a fused to pmaxGFP expressed in CTL to ask whether killing was inhibited. We reasoned that because the SHD of Slp1 and Slp2-a are 56% identical and 73% similar in their amino acid sequences, this SHD might inhibit both. We cannot rule out that this construct also inhibits other proteins that might bind Rab27a or other proteins involved in secretion by a similar SHD. However, to date, the SHD has only been identified in the Slps, and we have only detected expression of Slp1 and Slp2-a in CTL. It seems likely that there are other components to this complex as the SHD only gives a partial reduction in target cell killing, but which proteins these are remains to be determined.

## Materials and Methods

### Anti-Slp2-a SHD immunoglobulin G production

Recombinant glutathione S-transferase-tagged human Slp2-a SHD (corresponding to amino acids 1–99 of Slp2-a) was expressed in the DH5α*Escherichia coli* strain, harvested, purified and concentrated using an Amicon Ultra-15 centrifugal filter. Anti-Slp2-a SHD polyclonal serum was generated by Cambridge Research Biosciences after immunization with recombinant protein or generated as described in Imai et al. (37). Immunoglobulin G (IgG) was purified using a Protein A–Sepharose column (Pharmacia), and specificity was checked by Western blot and immunofluorescence on transfected cells.

### Cell culture

All murine CTLs were derived from C57BL/6 mice. Freshly isolated spleens were homogenized through a 70-μm strainer (Falcon) with the plunger from a 2-mL syringe. Splenocytes were washed once with Iscove's modified Dulbecco's medium (IMDM)/10% foetal calf serum (FCS) and cultured with an equal number of Balb/c splenocytes irradiated with 3000 rad in IMDM, 10% FCS, 100 U/mL recombinant interleukin-2, 55 μm 2-mercaptoethanol and 2 mm glutamine. After 5–7 days, CTLs were isolated over Histopaque (Sigma) and cultured for 2–5 days before use for staining, transfection or further rounds of stimulation. RBL cells were cultured in DMEM/10% FCS.

### Cloning and sequencing

The messenger RNA was isolated from cell pellets using a Qiagen RNeasy extraction kit. cDNA was generated using a Superscript II reverse transcriptase–polymerase chain reaction kit (Invitrogen). cDNA encoding murine Slp2-a was amplified by polymerase chain reaction (PCR) using Phusion high-fidelity polymerase (Finnzymes) with 5′ primer atg atc gac tta agt ttc ctg aca gag gag and 3′ primer tca ctt gga aag ctt ggc aat cag aag cat. cDNA encoding human Slp2-a was amplified by PCR using Pfu polymerase (Stratagene) with 5′ primer atc acc gcg ggc atg att gac tta agc ttc ctg act and 3′ primer aata ccc ggg tca ttt gga aat ctt ggc aat caa. These Slp2-a cDNAs were sequenced with overlapping primers to confirm sequence. A full-size fragment of murine Slp2-a fragment with *Sal* I and *Xma* I sites was generated by PCR with 5′ primer gatc gtc gac atg atc gac tta agt ttc ctg aca gag and 3′ primer gatc ccc ggg tca ctt gga aag ctt ggc aat cag aag, cut with the respective restriction enzymes and cloned into the pmaxFP-Green-C or -N vector (Amaxa). The dominant-negative murine Slp2-a construct (corresponding to amino acids 1–99) was generated by PCR with 5′ primer gcg aat tct atg atc gac tta agt ttc ctg aca g and 3′ primer gac tgg atc cct cat tgc tgt gtc ctt att ctg ctc and cloned into the pmaxFP-Green-N vector (Amaxa) at the *Bam* HI and *Eco* RI restriction sites. All constructs were confirmed by ABI sequencing before use.

### Lysates and Western blotting for Slp2-a

Cell pellets were washed once with PBS and lysed at 1 × 10^7^ cells/mL in 1% sodium dodecyl sulphate (SDS) containing complete protease inhibitor (Boehringer) by repeated passing through a 19G needle. The supernatant was centrifuged at 16 000 × ***g***for 10 min at 4°C to remove nuclei and membranes. Lysates were mixed with SDS loading buffer and heated to 95°C for 5 min prior to separation on a 7.5% SDS–PAGE. Proteins were transferred to a nitrocellulose membrane (Hybond; GE Healthcare) and blocked with PBS, 5% milk, 0.2% Tween-20 for 1 h at room temperature. Anti-Slp2-a SHD antibody was added in blocking buffer for at least 1 h, followed by three washes with PBS and 0.2% Tween-20. Horseradish peroxidase (HRP)-conjugated secondary antibody was incubated for 45 min in PBS, 5% milk, 0.2% Tween-20 and washed three times as above before development using Pierce Super Signal chemiluminescent reagent and Kodak Biomax MR film.

### Rab27a interactions

Mouse CTLs (2.5 × 10^7^ cells) were homogenized in a buffer containing 50 mm HEPES–KOH, pH 7.2, 150 mm NaCl, 1 mm MgCl_2_, 0.5 mm GTPγS and protease inhibitors (0.1 mm phenylmethylsulfonyl fluoride, 10 μm leupeptin and 10 μm pepstatin A) in a glass–Teflon Potter homogenizer by 10 strokes at 1000 r.p.m., and the proteins were solubilized with 1% Triton-X-100 at 4°C for 1 h. After removing the insoluble material by centrifugation at 17 400 × ***g***for 10 min, the supernatant was incubated overnight with a 30 μL volume of protein A–Sepharose beads (Amersham Biosciences) without a primary antibody for preabsorption. The supernatant was then incubated with anti-Slp1-C2B IgG (15), anti-Slp2-a-C2B IgG (37) or control anti-Slac2-c IgG (37) (10 μg/mL) for 1 h at 4°C followed by incubation with protein A–Sepharose beads for 1 h at 4°C. After washing the beads three times with 50 mm HEPES–KOH, pH 7.2, 150 mm NaCl and 0.2% Triton-X-100, the proteins bound to the beads were analyzed by 7.5–12.5% gradient SDS–PAGE and then immunoblotted with anti-Rab27a mouse monoclonal antibody (1/250 dilution; BD Transduction Laboratories), anti-Slp1 SHD (4 μg/mL), anti-Slp2-a SHD (4 μg/mL) and anti-Slac2-c rabbit polyclonal antibodies (4 μg/mL). SDS–PAGE and the immunoblot analysis were performed as described previously (37). Immunoreactive bands were visualized by enhanced chemiluminescence (Amersham Biosciences).

### Digestion of Slp2-a by proteases

Recombinant T7-tagged Slp1-5 proteins were expressed in COS-7 cells and purified with the anti-T7 tag antibody-conjugated agarose (Novagen) as described previously (38), but all procedures were performed in the absence of any protease inhibitors. Purified recombinant T7-Slp1–5 proteins were incubated at 25°C for 30 min with 100 ng/mL of trypsin. Digested proteins were then analyzed by 12.5% SDS–PAGE followed by immunoblotting with HRP-conjugated anti-T7 tag antibody as described previously (38). The immortalized melanocyte cell line melan-a (generous gift of Dorothy C. Bennett) was cultured as described previously (39). Melan-a cells (one 10-cm dish at confluence) were homogenized in 500 μL of a buffer (50 mm HEPES–KOH, pH 7.2, 1 mm MgCl_2_ and 150 mm NaCl) without any protease inhibitors in a glass–Teflon Potter homogenizer with 10 strokes at 1000 r.p.m., and proteins were solubilized with 1% Triton-X-100 at 4°C for 1 h. After removing the insoluble material by centrifugation at 15 000 r.p.m. for 10 min, cell lysates were incubated at 30°C for 1 h with either 2 mm EGTA or 750 μm Ca^2+^ in the presence or absence of calpain inhibitors (10 μm calpastatin peptide or 10 μm calpain inhibitor III; Calbiochem-Novachem Corp.). Reactions were terminated by adding SDS sample buffer and boiling for 3 min. Protein expression levels of Slp2-a and actin were analyzed by immunoblotting with specific antibodies as described previously (18).

### Confocal microscopy

CTLs were plated in serum-free medium onto glass slides for 15 min before complete media containing sera were added for a further 15 minutes. For conjugate formation, CTLs were mixed with equal numbers of P815 target cells before plating onto glass multi-well slides (Hendley). All cells were washed in serum-free RPMI in order to adhere them to glass for 15 min before adding back complete media containing serum. Cells were fixed with ice-cold methanol for 5 min, washed extensively with 1% BSA/PBS, followed by PBS, before mounting with PBS, and 90% glycerol containing 1,4, diazabicyclo(2,2,2)octane (DABCO). When counterstained with antibodies, cells were stained for 1 h at room temperature with primary antibody, washed extensively in 1% BSA/PBS and PBS prior to mounting. Primary antibodies used in this study were rabbit anti-cathepsin D (Upstate Biotechnology), -granzyme A, -Slp2-a SHD and rat anti-LAMP2, ABL93 (Developmental Studies Hybridoma Bank, Iowa). Secondary antibodies conjugated to fluorescein isothiocyanate or Cy-3 were obtained from Jackson Laboratories. Images were taken using a Zeiss Meta confocal microscope and processed using Zeiss LSM image browser (www.zeiss.com) and adobephotoshop.

### Transfection and cytotoxicity assay

RBL cells were transfected by electroporation as previously described (40). Three million human CD3 cells were stimulated with irradiated buffy coat cells in RPMI, 5% human serum, 2% interleukin-2 for 7 days prior to transfection with 10 μg Slp1 and Slp2-a DNA using the Amaxa nucleofector. About 2–4 × 10^7^ murine CTLs after two, three or four rounds of stimulation and 1–3 days post stimulation were nucleofected with 10 μg of DNA using an Amaxa nucleofector II, programme X-001 and the mouse T cell nucleofection kit according to the manufacturer's protocol. About 4 × 10^7^ murine CTLs, stimulated four times after isolation from spleen, were transfected with 10 μg Slp2-a constructs. The Slp2-a dominant-negative construct consisted of DNA encoding the first 100 amino acids of mouse Slp2-a fused to pmaxGFP at the carboxy terminus of Slp2-a. After 4.5 h, live cells were sorted to collect green cells (at fluorescence channel 1 (FL1) log >10^1^). Sorted cells were then used immediately to perform a killing assay using the Cytotox 96 assay (Promega) using P815 cells as targets.

## Supplementary Materials

Figure S1 Sequence of the splice variant of mouse Slp2-a used in these studies.

Movie S1 CTL derived from Rab27a–GFP mice (GFP, green and Lysotracker, red) showing Rab27a association with the membranes of secretory lysosomes. One CTL is forming an immunological synapse with a P815 target cell (unlabelled).

## References

[1] Stinchcombe JC, Bossi G, Booth S, Griffiths GM (2001). The immunological synapse of CTL contains a secretory domain and membrane bridges. Immunity.

[2] Stinchcombe JC, Majorovits E, Bossi G, Fuller S, Griffiths GM (2006). Centrosome polarization delivers secretory granules to the immunological synapse. Nature.

[3] Stinchcombe JC, Griffiths GM (2007). Secretory mechanisms in cell-mediated cytotoxicity. Annu Rev Cell Dev Biol.

[4] Menasche G, Pastural E, Feldmann J, Certain S, Ersoy F, Dupuis S, Wulffraat N, Bianchi D, Fischer A, Le Deist F, de Saint Basile G (2000). Mutations in RAB27A cause Griscelli syndrome associated with haemophagocytic syndrome. Nat Genet.

[5] Stinchcombe JC, Barral DC, Mules EH, Booth S, Hume AN, Machesky LM, Seabra MC, Griffiths GM (2001). Rab27a is required for regulated secretion in cytotoxic T lymphocytes. J Cell Biol.

[6] Feldmann J, Callebaut I, Raposo G, Certain S, Bacq D, Dumont C, Lambert N, Ouachee-Chardin M, Chedeville G, Tamary H, Minard-Colin V, Vilmer E, Blanche S, Le Deist F, Fischer A (2003). Munc13-4 is essential for cytolytic granules fusion and is mutated in a form of familial hemophagocytic lymphohistiocytosis (FHL3). Cell.

[7] Hume AN, Collinson LM, Rapak A, Gomes AQ, Hopkins CR, Seabra MC (2001). Rab27a regulates the peripheral distribution of melanosomes in melanocytes. J Cell Biol.

[8] Wu X, Rao K, Bowers MB, Copeland NG, Jenkins NA, Hammer JA (2001). Rab27a enables myosin Va-dependent melanosome capture by recruiting the myosin to the organelle. J Cell Sci.

[9] Munafo DB, Johnson JL, Ellis BA, Rutschmann S, Beutler B, Catz SD (2007). Rab27a is a key component of the secretory machinery of azurophilic granules in granulocytes. Biochem J.

[10] Kasai K, Ohara-Imaizumi M, Takahashi N, Mizutani S, Zhao S, Kikuta T, Kasai H, Nagamatsu S, Gomi H, Izumi T (2005). Rab27a mediates the tight docking of insulin granules onto the plasma membrane during glucose stimulation. J Clin Invest.

[11] Seabra MC, Coudrier E (2004). Rab GTPases and myosin motors in organelle motility. Traffic.

[12] Kuroda TS, Fukuda M (2004). Rab27A-binding protein Slp2-a is required for peripheral melanosome distribution and elongated cell shape in melanocytes. Nat Cell Biol.

[13] Yu M, Kasai K, Nagashima K, Torii S, Yokota-Hashimoto H, Okamoto K, Takeuchi T, Gomi H, Izumi T (2007). Exophilin4/Slp2-a targets glucagon granules to the plasma membrane through unique Ca^2+^-inhibitory phospholipid-binding activity of the C2A domain. Mol Biol Cell.

[14] Gomi H, Mizutani S, Kasai K, Itohara S, Izumi T (2005). Granuphilin molecularly docks insulin granules to the fusion machinery. J Cell Biol.

[15] Kuroda TS, Fukuda M, Ariga H, Mikoshiba K (2002). The Slp homology domain of synaptotagmin-like proteins 1-4 and Slac2 functions as a novel Rab27A binding domain. J Biol Chem.

[16] Fukuda M, Saegusa C, Mikoshiba K (2001). Novel splicing isoforms of synaptotagmin-like proteins 2 and 3: identification of the Slp homology domain. Biochem Biophys Res Commun.

[17] Wu XS, Rao K, Zhang H, Wang F, Sellers JR, Matesic LE, Copeland NG, Jenkins NA, Hammer JA (2002). Identification of an organelle receptor for myosin-Va. Nat Cell Biol.

[18] Fukuda M, Itoh T (2004). Slac2-a/melanophilin contains multiple PEST-like sequences that are highly sensitive to proteolysis. J Biol Chem.

[19] Dice JF (1987). Molecular determinants of protein half-lives in eukaryotic cells. FASEB J.

[20] Rechsteiner M, Rogers SW (1996). PEST sequences and regulation by proteolysis. Trends Biochem Sci.

[21] Rogers S, Wells R, Rechsteiner M (1986). Amino acid sequences common to rapidly degraded proteins: the PEST hypothesis. Science.

[22] Tolmachova T, Anders R, Stinchcombe J, Bossi G, Griffiths GM, Huxley C, Seabra MC (2004). A general role for Rab27a in secretory cells. Mol Biol Cell.

[23] Neeft M, Wieffer M, de Jong AS, Negroiu G, Metz CH, van Loon A, Griffith J, Krijgsveld J, Wulffraat N, Koch H, Heck AJ, Brose N, Kleijmeer M, van der Sluijs P (2005). Munc13-4 is an effector of rab27a and controls secretion of lysosomes in hematopoietic cells. Mol Biol Cell.

[24] Saegusa C, Tanaka T, Tani S, Itohara S, Mikoshiba K, Fukuda M (2006). Decreased basal mucus secretion by Slp2-a-deficient gastric surface mucous cells. Genes Cells.

[25] Fukuda M (2006). Rab27 and its effectors in secretory granule exocytosis: a novel docking machinery composed of a Rab27·effector complex. Biochem Soc Trans.

[26] Fukuda M (2006). Distinct Rab27A binding affinities of Slp2-a and Slac2-a/melanophilin: hierarchy of Rab27A effectors. Biochem Biophys Res Commun.

[27] Shirakawa R, Higashi T, Tabuchi A, Yoshioka A, Nishioka H, Fukuda M, Kita T, Horiuchi H (2004). Munc13-4 is a GTP-Rab27-binding protein regulating dense core granule secretion in platelets. J Biol Chem.

[28] Santoro A, Cannella S, Bossi G, Gallo F, Trizzino A, Pende D, Dieli F, Bruno G, Stinchcombe JC, Micalizzi C, De Fusco C, Danesino C, Moretta L, Notarangelo LD, Griffiths GM (2006). Novel Munc13-4 mutations in children and young adult patients with haemophagocytic lymphohistiocytosis. J Med Genet.

[29] Richmond JE, Weimer RM, Jorgensen EM (2001). An open form of syntaxin bypasses the requirement for UNC-13 in vesicle priming. Nature.

[30] Zhang B, Ginsburg D (2003). Getting secretory granules ready for prime time. Cell.

[31] Wojcik SM, Brose N (2007). Regulation of membrane fusion in synaptic excitation-secretion coupling: speed and accuracy matter. Neuron.

[32] Tsuboi T, Fukuda M (2006). The Slp4-a linker domain controls exocytosis through interaction with Munc18-1·effector syntaxin-1a complex. Mol Biol Cell.

[33] Fukuda M, Mikoshiba K (2001). Synaptotagmin-like protein 1-3: a novel family of C-terminal-type tandem C2 proteins. Biochem Biophys Res Commun.

[34] Goishi K, Mizuno K, Nakanishi H, Sasaki T (2004). Involvement of Rab27 in antigen-induced histamine release from rat basophilic leukemia 2H3 cells. Biochem Biophys Res Commun.

[35] Menager MM, Menasche G, Romao M, Knapnougel P, Ho CH, Garfa M, Raposo G, Feldmann J, Fischer A, de Saint Basile G (2007). Secretory cytotoxic granule maturation and exocytosis require the effector protein hMunc13-4. Nat Immunol.

[36] Martens S, Kozlov MM, McMahon HT (2007). How synaptotagmin promotes membrane fusion. Science.

[37] Imai A, Yoshie S, Nashida T, Shimomura H, Fukuda M (2004). The small GTPase Rab27B regulates amylase release from rat parotid acinar cells. J Cell Sci.

[38] Fukuda M, Kanno E, Mikoshiba K (1999). Conserved N-terminal cysteine motif is essential for homo- and heterodimer formation of synaptotagmins III, V, VI, and X. J Biol Chem.

[39] Kuroda TS, Ariga H, Fukuda M (2003). The actin-binding domain of Slac2-a/melanophilin is required for melanosome distribution in melanocytes. Mol Cell Biol.

[40] Bossi G, Griffiths GM (1999). Degranulation plays an essential part in regulating cell surface expression of Fas ligand in T cells and natural killer cells. Nat Med.

