# Supplementary material for: Slp1 and Slp2-a Localize to the Plasma Membrane of CTL and Contribute to Secretion from the Immunological Synapse
Source: Traffic. 2008 Feb 11;9(4):446–57. doi: 10.1111/j.1600-0854.2008.00714.x (PMC2329822; doi:10.1111/j.1600-0854.2008.00714.x)
Supplement: Figure S1 — Sequence of the splice variant of mouse Slp2-a used in these studies. [file tra0009-0446-SD1.doc]

**Supplementary information. (Holt et al)**

Nucleotide and protein sequence of mouse Slp2a cDNA from CTL, used in this study.

1 atgatcgacttaagtttcctgacagaggaggagcaagacgccatcttaaaggttctgcag

1 M I D L S F L T E E E Q D A I L K V L Q

61 agggatgctgccctgaagagggctgaagaggagagagtcagacatctgcctgaaaaaatt

21 R D A A L K R A E E E R V R H L P E K I

121 aaggatgaccaacaactaaagaatatgagtggccaatggttttatgaagctaaggcaaaa

41 K D D Q Q L K N M S G Q W F Y E A K A K

181 agacacagggacaaaatccacggtgctgacatcatcagagcatccatgagaaggaagaag

61 R H R D K I H G A D I I R A S M R R K K

241 ctcccagcggcagctgagcagaataaggacacagcaatgagggcaaaggagagctgggtg

81 L P A A A E Q N K D T A M R A K E S W V

301 aataatgttaacaaagatgctgtccttcctccagagatagctgttgtggaagagccagaa

101 N N V N K D A V L P P E I A V V E E P E

361 gatgacacagatcccgcaggcccaagttccagtttggtggatccagcttccagtgtgatt

121 D D T D P A G P S S S L V D P A S S V I

421 gatatgtcccaggaaagtacgaggactccagctgtgtctctgcccaagcaaaggaagaat

141 D M S Q E S T R T P A V S L P K Q R K N

481 ccattcaacagccccaagctgccggaagatcactccttgcaacaaaccaaacccgagcag

161 P F N S P K L P E D H S L Q Q T K P E Q

541 tcaaaaactggaaaagctggtttatttcagatttcaaaagagggtgagttgtcagaatca

181 S K T G K A G L F Q I S K E G E L S E S

601 aaagaaaagtcatctatcccagatatgccacgtcagcagttagagaaaccaaagcaaact

201 K E K S S I P D M P R Q Q L E K P K Q T

661 gtatccacagagcctgaaaatgcgtctcataccaaagctccaatccccaaagccagaaaa

221 V S T E P E N A S H T K A P I P K A R K

721 ctgatctacaaatcaaatgacttagagaaagatgataaccagtcttttcccagacaacgg

241 L I Y K S N D L E K D D N Q S F P R Q R

781 agggactccctgaatgcgagaggggctccaagagggatcctaaagcgcaactccagttcc

261 R D S L N A R G A P R G I L K R N S S S

841 agcagcacggactcagagaccctccgtttaaattacaaccttgatccgaaaagcaaaatc

281 S S T D S E T L R L N Y N L D P K S K I

901 ctatcacctggcctaaccatccacgagagaatctctgagaaggaattttctttagaagac

301 L S P G L T I H E R I S E K E F S L E D

961 gactcttccacaagctcactagagccgttaaagcacgtgagattctctgccgtgaagaat

321 D S S T S S L E P L K H V R F S A V K N

1021 gagcttccacaaagtcctagacccgtccttggccaggaagtgggagaatttactgtttta

341 E L P Q S P R P V L G Q E V G E F T V L

1081 gaatctgaccagttgcaaaatggaactgaagatgcaggggacatagaggagtttcagaat

361 E S D Q L Q N G T E D A G D I E E F Q N

1141 cacccagagctctcccacaaaacacctttgtctcattatcagttagtgtcaagcccaagc

381 H P E L S H K T P L S H Y Q L V S S P S

1201 gattcaggaagggaaagagaacagctgatgtcttctgggtctgctccaagagatgagatc

401 D S G R E R E Q L M S S G S A P R D E I

1261 ccttgtcattcagacattctacccacaggacctcagtgtgttgagagttcatccgtcatc

421 P C H S D I L P T G P Q C V E S S S V I

1321 aatgggcaacaagagaaatcatcacattttacaaagcttccatcagaattgtccaaaagc

441 N G Q Q E K S S H F T K L P S E L S K S

1381 ccttccgatgaactgactcagtgtggtgagcctgagccatcacagacagcagaccacagt

461 P S D E L T Q C G E P E P S Q T A D H S

1441 tttagagaccatcggcaaggttcagaagaagagcacagccctgttttgaaaactttggaa

481 F R D H R Q G S E E E H S P V L K T L E

1501 agaagagctgctaggaaattgccttccaaaagtctagaagacattccatcagattcatca

501 R R A A R K L P S K S L E D I P S D S S

1561 aatcaagcaaaagtagataatctgcctgaagaattagtgcgtagtgctgaagatgtttcc

521 N Q A K V D N L P E E L V R S A E D V S

1621 acagtgccttcactgcctgataatcagttttcccaccctgacaaactcaaaaggatgagc

541 T V P S L P D N Q F S H P D K L K R M S

1681 aagtccgtgccagcatttcttcaagatgaggcgagtggcagtgtgatgagcgtttacagt

561 K S V P A F L Q D E A S G S V M S V Y S

1741 ggagactttggcaacctagaagtgaaaggaagcgtgcagtttgcactcgactacgtggag

581 G D F G N L E V K G S V Q F A L D Y V E

1801 tccctgaaagagctgcatgtgtttgtggcccagtgtaaggatttagcagcagcagatgtt

601 S L K E L H V F V A Q C K D L A A A D V

1861 aagaaacagcgctcagatccgtatgtaaagacctatctgctaccagacaaaggcaaaatg

621 K K Q R S D P Y V K T Y L L P D K G K M

1921 ggcaagaagaagacactcgtagtgaagaagaccttgaatcctgtatacaacgagatattg

641 G K K K T L V V K K T L N P V Y N E I L

1981 cggtataaaattgaaaggcaattcttaaagacgcagaagttgaacctgtccgtttggcat

661 R Y K I E R Q F L K T Q K L N L S V W H

2041 cgggatacatttaagcgcaacagctttctgggggaggtggagctcgacctggaaacgtgg

681 R D T F K R N S F L G E V E L D L E T W

2101 gattgggacagcaaacagaacaaacagctgaagtggtacccactgaagaggaagacagca

701 D W D S K Q N K Q L K W Y P L K R K T A

2161 ccagttgccctcgagacagaaaacagaggtgaaatgaaactagctctccagtatgttccg

721 P V A L E T E N R G E M K L A L Q Y V P

2221 gaaccaagccctggcaaaaagcttcctacaactggagaagtccacatctgggtgaaggaa

741 E P S P G K K L P T T G E V H I W V K E

2281 tgccttgacctcccactgttgaggggcagccacctaaattcttttgttaaatgtaccatc

761 C L D L P L L R G S H L N S F V K C T I

2341 cttccagataccagtagaaaaagtcgccagaagacaagagctgtagggaaaaccaccaac

781 L P D T S R K S R Q K T R A V G K T T N

2401 cccgtcttcaaccataccatggtgtatgatgggttcaggcctgaagatctgatggaagcc

801 P V F N H T M V Y D G F R P E D L M E A

2461 tgtgtagaactcacagtctgggaccattataaactaaccaaccagtttctgggaggtctc

821 C V E L T V W D H Y K L T N Q F L G G L

2521 cggatcggctttggaacaggaaaaagctacgggactgaagtggattggatggattctact

841 R I G F G T G K S Y G T E V D W M D S T

2581 tctgaggaagttgctctctgggagaagatggtaaactctcccaacacttgggttgaagcg

861 S E E V A L W E K M V N S P N T W V E A

2641 acgctgcccctccggatgcttctgattgccaagctttccaagtga 2685

881 T L P L R M L L I A K L S K * 894
