# Supplementary figures and images for: Slp1 and Slp2-a Localize to the Plasma Membrane of CTL and Contribute to Secretion from the Immunological Synapse
Source: Traffic. 2008 Feb 11;9(4):446–57. doi: 10.1111/j.1600-0854.2008.00714.x (PMC2329822; doi:10.1111/j.1600-0854.2008.00714.x)

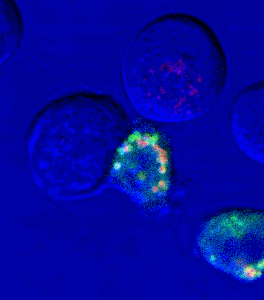

Supplement: Movie S1 — CTL derived from Rab27a–GFP mice (GFP, green and Lysotracker, red) showing Rab27a association with the membranes of secretory lysosomes. One CTL is forming an immunological synapse with a P815 target cell (unlabelled). [file tra0009-0446-SD2.gif]
